# Epidemiology and Genetic Diversity of Rotavirus Strains in Children with Acute Gastroenteritis in Lahore, Pakistan

**DOI:** 10.1371/journal.pone.0067998

**Published:** 2013-06-25

**Authors:** Muhammad Masroor Alam, Adnan Khurshid, Shahzad Shaukat, Rana Muhammad Suleman, Salmaan Sharif, Mehar Angez, Salman Akbar Malik, Tahir Masood Ahmed, Uzma Bashir Aamir, Muhammad Naeem, Syed Sohail Zahoor Zaidi

**Affiliations:** 1 Department of Biotechnology, Quaid-i-Azam University, Islamabad, Pakistan; 2 Department of Virology, National Institute of Health, Islamabad, Pakistan; 3 Department of Biochemistry, Quaid-i-Azam University, Islamabad, Pakistan; 4 Department of Pediatrics, The Children’s Hospital Lahore, Lahore, Pakistan; Centers for Disease Control and Prevention, United States of America

## Abstract

Pakistan harbors high disease burden of gastro-enteric infections with majority of these caused by rotavirus. Unfortunately, lack of proper surveillance programs and laboratory facilities have resulted in scarcity of available data on rotavirus associated disease burden and epidemiological information in the country. We investigated 1306 stool samples collected over two years (2008–2009) from hospitalized children under 5 years of age for the presence of rotavirus strains and its genotypic diversity in Lahore. The prevalence rate during 2008 and 2009 was found to be 34% (n = 447 out of 1306). No significant difference was found between different age groups positive for rotavirus (*p*>0.05). A subset of EIA positive samples was further screened for rotavirus RNA through RT-PCR and 44 (49.43%) samples, out of total 89 EIA positive samples, were found positive. G and P type prevalence was found as follows: G1P [4] = 3(6.81%); G1P [6] = 9(20.45%); G1P [8] = 1(2.27%); G2P [4] = 21(47.72%); G2P [8] = 1(2.27%); G9P [4] = 1(2.27%); G9P [6] = 1(2.27%) and G9P [8] = 7(15.90%). Phylogenetic analysis revealed that the VP7 and VP4 sequences clustered closely with the previously detected strains in the country as well as Belgian rotaviruses. Antigenic characterization was performed by analyzing major epitopes in the immunodominant VP7 and VP4 gene segments. Although the neutralization conferring motifs were found variable between the Pakistani strains and the two recommended vaccines strains (Rotarix™ and RotaTeq™), we validate the use of rotavirus vaccine in Pakistan based on the proven and recognized vaccine efficacy across the globe. Our findings constitute the first report on rotavirus’ genotype diversity, their phylogenetic relatedness and epidemiology during the pre-vaccination era in Lahore, Pakistan and support the immediate introduction of rotavirus vaccine in the routine immunization program of the country.

## Introduction

Diarrheal infections are considered as a significant cause of infant and childhood morbidity and mortality in both developing as well as developed countries [1]. Most of these infections have a viral etiology principally rotaviruses that alone account for approximately 527,000 (475000–580000) deaths per annum among children less than 5 years of age [2], [3]. Globally, more than 2 million children are hospitalized every year for rotavirus infection and 90% of rotavirus associated mortalities occur in Africa and Asia [4], [5]. Rotaviruses have been classified into seven heterogenic groups (A to G) based on their genetic and antigenic properties. Group A rotaviruses are the most important and frequently detected viruses among the three groups A, B and C that infect humans [6]. The virus belongs to the family *reoviridae* with triple-coated icosahedral virion particle, enclosed within is the 11 segmented double stranded RNA genome. The triple layered capsid consists of 2 proteins in the outer shell (VP7 and VP4), the intermediate layer constitutes VP6 while VP2 forms the inner layer enclosing two proteins VP1 and VP3. Based on VP6 capsid gene, the virus has been classified into seven major genogroups while VP7 and VP4 are the basis of a binary-system for further classifying the genogroup A viruses into 27 G- and 35 P- genotypes respectively [7]. Globally, the most important types causing majority of infections are G1P [8], G2P [4], G3P [8], G4P [8] and G9P [8] [8], [9]. However, significant diversity of rotavirus genotypes have emerged with several novel combinations due to accumulation of point mutations, genome re-assortments or zoonotic transmission of animal strains resulting in the introduction of new antigenic variants [10], [11].

Rotavirus strain identification is considered as the key component of epidemiological surveys, disease distribution, genotype prevalence studies, vaccine administration and efficacy monitoring programs. Many past studies have highlighted the significance of continued monitoring of circulating rotavirus strains in order to maintain sufficient population immunity [12]. In Pakistan, there is no well-developed surveillance system for rotavirus strain identification although country’s Ministry of Health has initiated a hospital based surveillance network to serologically test the stool samples from children presented with gastroenteritis at central district hospitals in 3 major cities; Karachi (Sindh province), Lahore and Rawalpindi (Punjab province). These sentinel sites perform ELISA for the diagnosis of rotavirus infection without any further analysis for viral genotype identification. This study is in continuation to our previous work where we identified an emerging rotavirus genotype G12 in two children admitted to a hospital in Rawalpindi [13]. To further explore the epidemiology and genotypic diversity of rotavirus in Pakistan, here we report the findings of rotavirus subtypes detected in children hospitalized, due to severe dehydrating diarrhea, at Children’s Hospital Lahore.

## Materials and Methods

Samples from hospitalized children suspected of rotavirus gastroenteritis were collected as per World Health Organization’s standard case definitions that describe a suspected case as a child <5 years of age, admitted to a designated sentinel hospital for treatment of gastroenteritis while a confirmed case is a suspected case in whose stool the presence of rotavirus is demonstrated by means of an enzyme immunoassay. The study concept and design was approved through the Pakistan’s National Institute of Health Internal Review Board. The samples were collected after informed and written consent from the patient’s parents/guardians.

A total of 1306 stool samples were collected from hospitalized patients at ‘The Children’s Hospital’ Lahore during January 2008 to December 2009. Majority of these samples were collected from children below than 5 years of age who were hospitalized with suspected rotavirus gastroenteritis. The collected stool samples were processed for the detection and confirmation of Rotavirus antigen. ELISA test was performed using the ProSpecT™ Rotavirus Microplate Assay (Oxoid Ltd., Basingstoke Hants, UK) as per World Health Organization’s recommendations (http://whqlibdoc.who.int/publications/2011/9789241502641_eng.pdf).

A subset (20%) of EIA positive samples were transported to Department of Virology, National Institute of Health, Islamabad for rotavirus RNA detection through RT-PCR and further genotype determination on the basis of VP7 and VP4 gene segments using the protocol as described by Gouvea et al 1990 [14] and Gentsch et al 1992 [15] respectively. Amplified products from round 1 PCR reactions were purified using QIAquick PCR purification kit (Qiagen, Germany) and were directly sequenced for VP7 and VP4 genes using the Big dye terminator sequencing kit v3.0 by automated Genetic analyzer ABI 3130xl (Applied Biosystems).

Phylogenetic analyses of VP7 and VP4 sequences were performed in comparison to the strains belonging to different geographical regions as retrieved from GenBank. Evolutionary tree and distances (number of base substitutions per site) were generated by Neighbor Joining method with Kimura-2 parameter using MEGA 4.0 (http://megasoftware.net/). The percentage of replicate trees in which the associated taxa clustered together in the bootstrap test (1000 replicates) is shown next to the branches. The GenBank accession numbers, country, year of sample collection and respective genotype information has been given where available. The VP7 sequences obtained in this study have been submitted to GenBank under the accession numbers KC896141-KC896157 and the VP4 sequences under accession numbers KC896127-KC896140.

## Results

### Prevalence, Epidemiology and RVA-Genotypes

**Figure 1:**
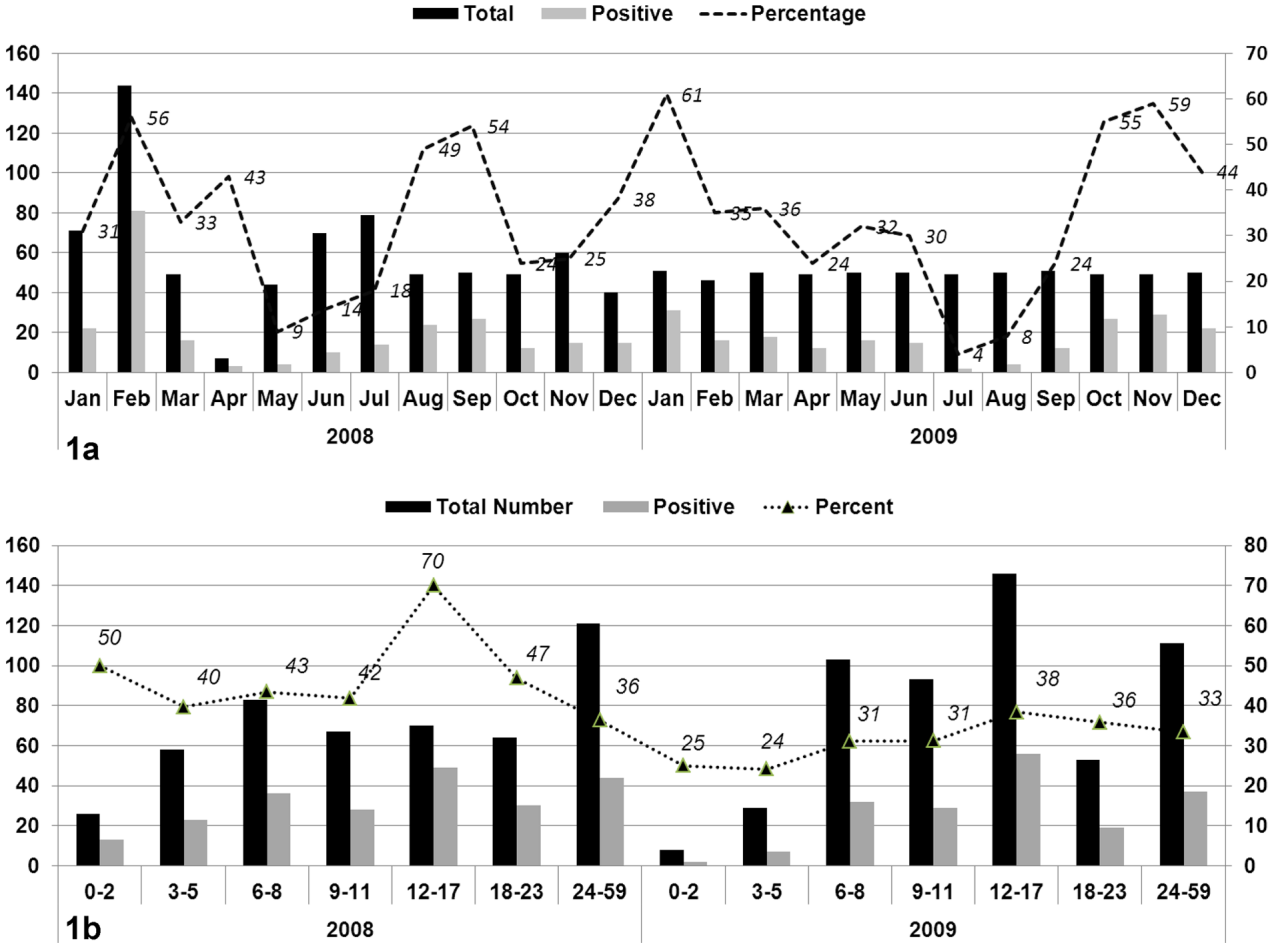
Phylogenetic analysis of VP7 gene segment of the study viruses isolated in hospitalized children at Lahore. Each of the G1, G2 and G9 genotype viruses from this study are indicated with round circle, while the previous available sequences in GenBank from Pakistan have been given with an arrow-head. The closely matched sequences have retrieved from NCBI GenBank and included to reconstruct a phylogenetic tree (Figure 1a). Phylogenetic analysis of VP4 gene segment of the study viruses isolated in hospitalized children at Lahore. Each of the P [4], P [6] and P [8] genotype viruses from this study are indicated with round circle, while the previous available sequences in GenBank from Pakistan have been given with an arrow-head. The closely matched sequences have retrieved from NCBI GenBank and included to reconstruct a phylogenetic tree (Figure 1b).

Screening of total 1306 samples collected during two years period (2008–2009) for the presence of rotavirus antigen yielded 447 (34.22%) samples positive for group A major inner capsid protein (VP6 antigen) common to all known rotavirus genotypes. The prevalence rates during 2008 and 2009 were 34.12% (n = 243 out of 712) and 34.34% (204 out of 594) respectively. No significant difference (*P*>0.05) was found between different age groups positive for rotavirus through ELISA; however, high infection rate was observed among children between 12–17 and 24–59 months during 2008 and 2009 respectively. The rotavirus positive cases appeared to occur throughout the year with peaks during January to April and July to September (Figure 1a and 1b).

A subset of EIA positive samples, based on availability, was further screened for the presence of rotavirus RNA through RT-PCR. Out of total 89 EIA positive samples available from archived stock kept at -80°C, 44 (49.43%) samples were found positive for rotavirus through PCR. G and P types were found with the prevalence as G1P [4] = 3(6.81%); G1P [6] = 9(20.45%); G1P [8] = 1(2.27%); G2P [4] = 21(47.72%); G2P [8] = 1(2.27%); G9P [4] = 1(2.27%); G9P [6] = 1(2.27%) and G9P [8] = 7(15.90%).

### Phylogenetic Analysis Based on RVA-VP7 and -VP4 Gene Segments

The genetic sequence of 18 viruses included in this study was determined for the VP7 gene. These samples were randomly selected on the basis of sample quantity and resource limitations which showed that all viruses are highly identical (99–100%) to each other within their respective genotypes; G1, G2 and G9. The G1 strains shared close sequence similarity with the previously reported viruses from Pakistan (JN001862) as well as to those from Belgium (HQ392261, HQ392204) and USA (JN258346). The VP7 sequences of G9 strains were found 100% identical among themselves as well as highly similar level of homology was found with the viruses from South Africa (GQ338887), Russia (FJ447573) and Australia (AY307090). These viruses also hold 98% similarity to previously reported G9 strains (JN001865, JN001866) from Faisalabad city of Pakistan (unpublished data). Likewise, the G2 viruses showed 99–100% identity among themselves and a similar genetic closeness was found among the G2 strains from Bangladesh (EF690778, EF690782) as well as the already identified G2 strains from Pakistan (JN001883; unpublished data).

The genetic relationships of rotavirus strains from this study were also determined on the basis of VP4 gene. 14 viruses were grouped among three genotypes P [4], P [6] and P [8] with the highest prevalence of P [8] in combination with G1, G2 and G9. The genotype P [6] was found with G1 and G9 counterparts while P [4] was found with G2 as the most common circulating strains during the study period with 48% prevalence. In a pattern similar to the G-types, P [6] and P [8] strains detected from Lahore during 2008 and 2009 revealed closest identity to already reported viruses from Pakistan (JN001876-79 unpublished data).

**Figure 2:**
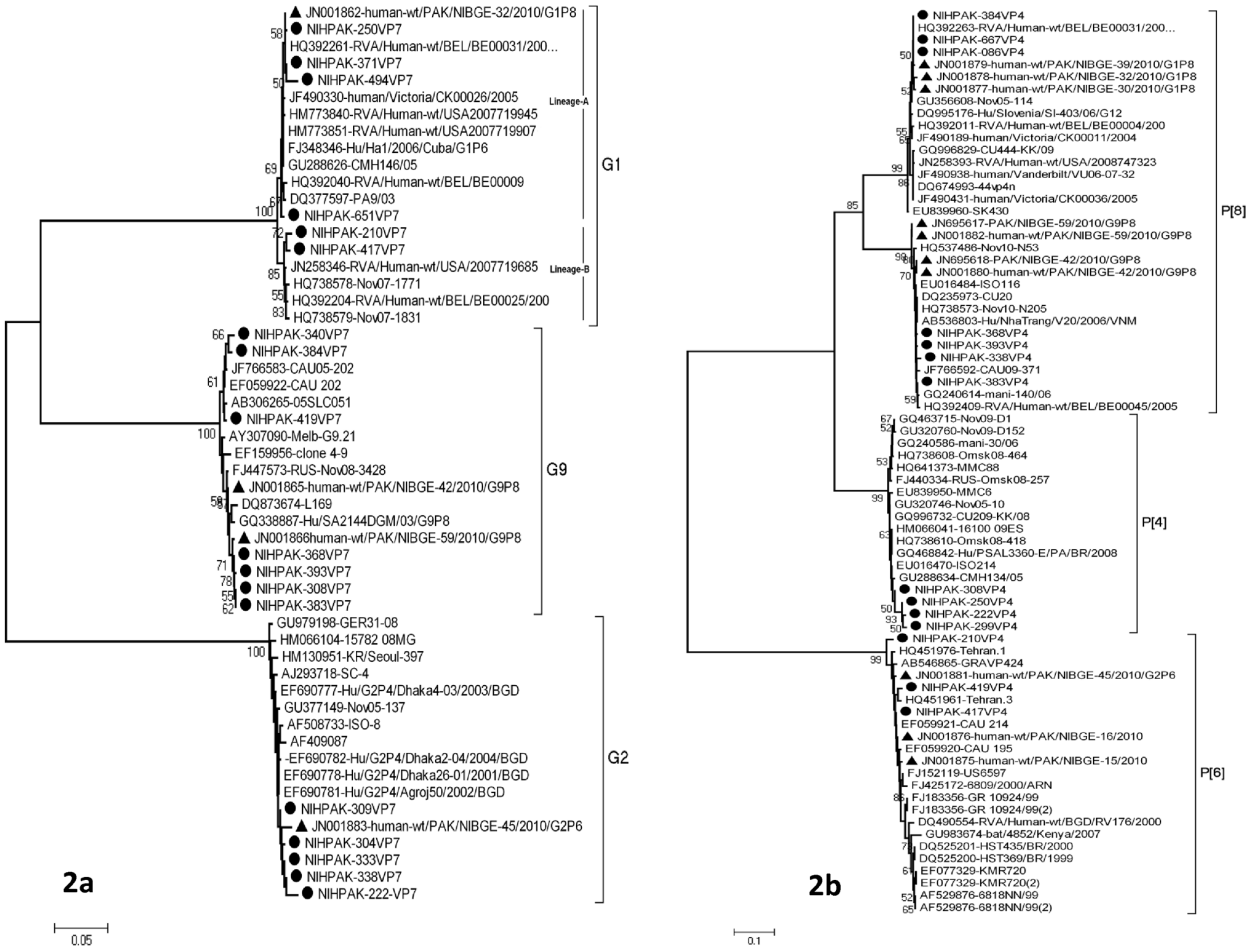
Number of samples collected from hospitalized children admitted with gastroenteritis during January 2008 to December 2009. Months are represented on X-axis while Y-axis indicates Number of cases. The percentages are shown on secondary axis. Total, Positive and percentage figures are represented by black, grey and dotted line trend respectively (Figure 2a). Age wise distribution of samples collected from hospitalized patinets presented with the complaints of gastroenteritis during January 2008 to December 2009. The total number of samples were distributed among age-groups (given on X-axis) while number of samples are given on Y-axis (total no. shown in black and EIA psoitive samples in grey color) with the percentages shown with data labels (Figure 2b).

Phylogenetic reconstruction revealed clustering of all rotavirus strains from Children Hospital, Lahore with viruses (JN001862 (G1P [8]); JN001865 (G9P [8]); JN001883 (G2P [6]) previously detected from the Faisalabad district, located in the South-West of Lahore at a distance of 118 kilometers (Figure 2a and 2b). The viruses classified as G1 are categorized into two separate lineages. Lineage A contains rotavirus strains from both Lahore and Faisalabad. Two of the strains (NIHPAK-210 and NIHPAK-417) were distinguished as Lineage B clustering with viruses from USA (JN258346) and Belgium (HQ392204).

### Comparison of RVA-VP7 Antigenic Regions between Pakistani and Vaccine Strains

**Figure 3:**
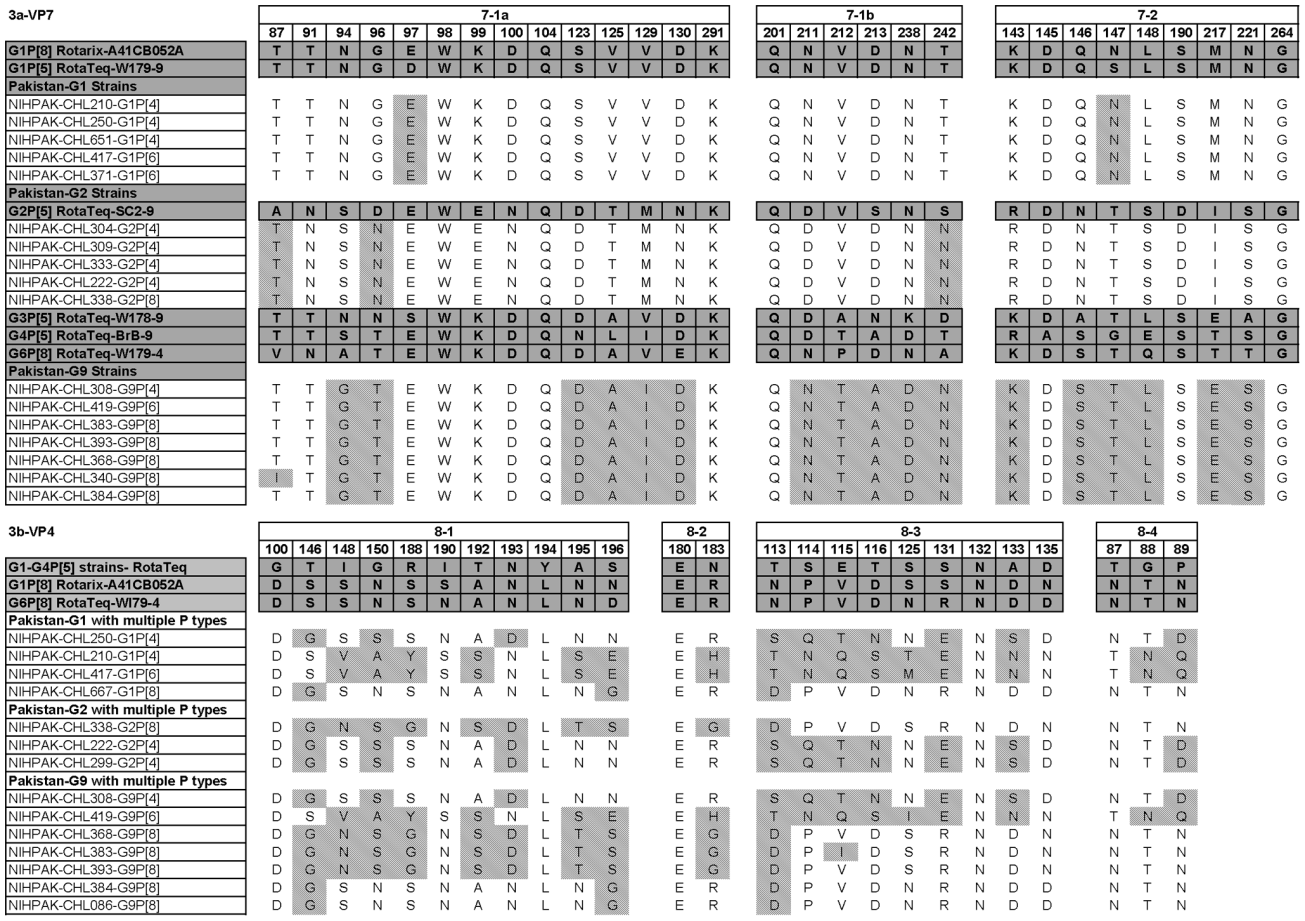
Alignment and comparison of surface exposed amino acid motifs constituting the major epitopes of the rotavirus strains. Antigenic regions of VP7 (Figure 3a) and VP4 (Figure 3b) proteins were aligned and compared between the Pakistani rotavirus strains and those of the two licensed vaccines; Rotarix™ and RotaTeq™. The differences are highlighted by the shaded positions. These differences indicate a variation in comparison either with one of the two vaccines. Antigenic regions within the VP7 are shown above as 7-1a, 7-1b and 7-2. For VP4, the antigenic regions are indicated as 8-1, 8-2, 8-3 and 8-4.

The effectiveness of rotavirus vaccines can be determined by analyzing the amino acid (aa) differences, in the neutralizing epitopes of VP7 and VP4 proteins, between vaccine and the circulating strains. We compared the VP7 aa sequences of Pakistani viruses, constituting the antigenic epitopes with those of the two available vaccines strains (RotaTeq™ and Rotarix™). The VP7 protein contains three antigenic epitopes defined as 7-1a, 7-1b and 7-2 which comprise of 29 amino acid residues from positions 87 to 291 based on rhesus Rotavirus strain numbering (GenBank accession No. AF295303) [16]. The comparison of all Pakistani rotavirus strains found in Lahore and vaccine strains showed that out of total 29 amino acids, only five are conserved (Figure 3a).

Analysis of Pakistani G1 strains showed that the 29 amino acid residues across the three antigenic sites are highly conserved when compared to Rotarix™ G1P [8] strain while 02 differences were found at positions 97 (D>E; Aspartic acid to Glutamic acid) and 147 (S>N; Serine to Asparagine) within the 7-1a and 7-2 regions when compared to RotaTeq™ G1P [5] strain.

The VP7 antigenic epitopes of G2 Pakistani viruses showed 3 variations (02 substitutions within 7-1a and 01 change in 7-1b) from the RotaTeq™ G2P [5] strain. These differences were observed at positions 87 and 96 of 7-1a epitopes (A>T; Alanine to Threonine and D>N; Aspartic acid to Asparagine) and 242 in 7-1b (S>N; Serine to Asparagine) while the aa residues within the 7-2 epitope were all conserved. When compared to Rotarix™ strain, 18 variations were noticed spanning across the three epitope regions, predominantly in the 7-1a and 7-2 regions.

Among the G9 viruses presented in this study, the VP7 protein was compared with Rotarix™ G1 strain as well as all constituent strains of RotaTeq™ vaccine (G1-4 with P [5] and G6P [8]). Thirteen aa changes were found when compared to Rotarix™ strain across all three epitopes. On comparison with the RotaTeq™ G1 strain, 14 variations were found in Pakistani viruses. In comparison to G2 RotaTeq™ strain, 19 aminoacid changes were noticed. Similarly, 11 variations were observed between G3 RotaTeq™ strain and the Pakistani G9 viruses with majority of differences found in 7-1b epitope in which all the aa residues were changed except Glutamine at position 201. For G4 RotaTeq™ strain, 10 aa changes were found in study strains across the three epitope regions. When compared to G6P [8] RotaTeq™ strain, 12 dissimilarities were noticed across the three antigenic regions mainly within the 7-1b region.

### Comparison of RVA-VP4 Antigenic Regions between Pakistani and Vaccine Strains

The VP4 protein of rotavirus contains four antigenic regions defined as 8-1 to 8-4 spanning between aminoacid positions 88 to 196 based on rhesus Rotavirus strain (GenBank accession No. AY033150) [17]. The VP4 sequences of Pakistani viruses were compared to those of vaccine strains and found significant variations among the four regions except aminoacid residue 180 and 132 which were well conserved amongst all Pakistani and vaccine strains (Figure 3b).

When compared to Rotarix™ P [8] strain, the Pakistani P [8] strains with G1 and G2 counterparts showed higher degree of disparity than those with G9. Importantly, the Pakistani strains contained 3 to 12 residues that differed from the RotaTeq™ P [8] strain while Rotarix™ strain P [8] was more variable with 7 to 13 aminoacid differences. Interestingly, all the three aminoacid residues (87–89; NTN) of 8-4 epitope were well conserved among all P [8] Pakistani viruses.

A high degree of variation was observed for P [4] and P [6] strains as well. For P [4] strains, there were 13 aminoacid differences when compared to Rotarix™ while 22 to 23 residues were different from RotaTeq™ strains. Comparison of P [6] strains showed 17 differences with Rotarix™ whereas 20 aminoacid differences were found when compared to RotaTeq™ strains.

## Discussion

Diarrhea is accountable for approx. 5% of all deaths among children below five years of age. Viral pathogens are the most common cause of gastroenteritis in both developing and developed countries [18]–[20] especially rotaviruses, noroviruses, astroviruses and adenoviruses [21]. Three rotavirus serogroups (A, B and C) are known to cause gastroenteritis in humans [22]. The most commonly associated VP7 genotypes of group-A rotavirus with different combinations of VP4 genetic counterparts are G1, G2, G3, G4 and G9 [23]. However, unusual genotypes with different G and P specifities have recently been reported from various parts of the world [1].

Seasonality of rotavirus gastroenteritis in Pakistan has never been determined although the cases have been detected round the year [24]. In our study, rotavirus was found during all seasons especially early and late calendar months. This may be attributed to the rainy and post-monsoon season in our country and support the previous findings that low air temperature and dry environment increase the rotavirus incidence [25]. The same pattern is peculiar to tropical areas where rotavirus infections appear throughout the year without any significant epidemic peaks [26]. Similar findings have been reported from Bangladesh, South Asian regions, Bahrain and Costa Rica where rotavirus is detected all year round with less obvious seasonality [27]–[30]. However, our findings contrast those in USA, Japan, Northern Asian region, Australia and Europe where rotavirus diarrhea peaks in winter and is rarely identified in summer [31]–[35].

The higher percentage of samples positive for rotavirus was found among the children between 12 and 17 months of age. These findings are slightly variable than the previous study conducted in Karachi city of Pakistan [36] where most of the rotavirus infections were found among children less than 12 months of age. This variation might reflect a different study design and target population i.e. our study includes the samples from patients hospitalized with severe gastroenteritis whereas Qazi *et al.* [36] studied the incidence of rotavirus associated infections in low-income communities of Karachi.

During the last 15 years, very few reports about circulating genotypes of rotavirus are available from Pakistan [36]–[38] in which circulation of G1, G2, G4 and G9 in combination with P [4], P [6], P [8], P [11] have been described. The current study is focused on the detection of circulating genotypes of rotavirus causing gastroenteritis in Lahore city of Punjab province and provides relatively comprehensive epidemiological as well as genetic information. Although G1 is the most common genotype found in diarrhea or gastroenteritis cases worldwide, we found G2P [4] as the most prevalent genotype in 48% of study samples. Genotype G9 was found among 20% of our samples in combinations with P [4], P [6] and P [8]. A similar increased prevalence of G9 strains have been found in neighboring countries including India and Bangladesh [39]. This genotype has recently gained sufficient epidemiological concern worldwide due to its variable vaccine response and infectivity rate [9], [40]. We found G9P [8] as the third most prevalent genotype which is consistent with the recent findings from India [41]. 20% of our samples were found positive for G1P [6] which has not been reported previously from Pakistan. We did not detect any G3 and G4 genotypes in our samples which substantiate the global scenario of their significant decline during the recent years [41]. G1P [8] was found in only 1 sample even though it has remained the most common strain detected globally and constitutes one of the currently recommended vaccine, Rotarix™. Surprisingly, we did not find any G12 genotype in this study which has recently emerged with numerous reports of global spread [42], [43]. We cannot speculate the absolute absence of this genotype from our population due to limited time period and number of samples selected for this study. Such variation and vast genetic differences of rotavirus strains evoke the need for continued surveillance to assess and consider the role of strain variability in the design of new vaccine candidates as well as to measure the impact of vaccine introduction in the community. The continued surveillance programs and characterization of rotavirus strains during the post-vaccination era is needed to monitor the emergence of ‘escape’ strains due to long-term pressure exerted by homotypic immunity [44].

In the present study, we compared the amino acid motifs constituting the neutralizing epitopes of VP7 and VP4 proteins between the circulating Pakistani rotaviruses and available vaccine strains. Multiple antigenic variations were noticed among both VP7 and VP4 epitopes highlighting the need for detailed antigenic mapping of prevalent rotavirus genotypes in the country. Complete genome sequencing will therefore be required to generate more comprehensive information on molecular epidemiology and evolutionary dynamics of rotaviruses on account of its segmented genome and the possible role of internal genes in immunity [45]. Although the aminoacid residues within the VP7 protein of G1 strains showed higher similarities with the Rotarix™ strains, significant differences were found amongst all four epitopes of VP4 proteins. In addition, a comparable degree of disparities was noticed among the RotaTeq™ and the Pakistani strains in both VP7 and VP4 proteins. Although such differences do not change the genotype specificity of the rotavirus strain, they may significantly influence the binding of neutralizing antibodies and hence viral fitness through selective pressure [45]. Similar findings have been reported from countries such as Belgium where rotavirus immunization program was initiated in 2006 followed by rapid massive coverage of up to 88% [46].

The vaccine introduction has significantly reduced the rotavirus disease burden in many countries like Australia, Austria, Belgium, Brazil, El Salvador, Nicaragua, Panama and the United States and both vaccines have been found to carry equivalent efficacy against G1, G3, G4 and G9 strains with P [8] specificity [47]. The Rotarix™ vaccine efficacy against G2 strains is somewhat lower as compared to other genotypes while data pertaining to G12 strains is not available as yet [47]. In addition, the vaccine introduction has modified the epidemiological patterns of circulating strains in certain regions such as the upsurge of G3 strains in the United States and Australia after introduction of RotaTeq™ [48]–[50]. Therefore, further detailed epidemiological studies must be planned to monitor the vaccine response during pre-and post-vaccination periods in Pakistani Population.

The mutations in the antigenic regions of rotavirus play an important role in the outcome of vaccine response. When compared to the respective prototype strains, our data substantiates the previous findings [51], [52] that the currently circulating G9 genotypes are different from their prototypes that were isolated during 1980s. There are about 15 known mutations in the antigenic regions of G9 genotype that can modify the antigenicity of the respective region [53]. Similarly, for G1 genotypes, the substitutions at positions 94, 97, 147 and 291 of VP7 protein are found to play a significant role in antigenic recognition [54]. In respect to G2 rotavirus genotypes, the substitution D96N within the antigenic region A was found responsible for failure of G2- specific monoclonal antibodies to react [55], but none of these mutations were found in Pakistani strains, except for G2 strains where D96N; Aspartic acid to Asparagine, substitution was found amongst all viruses from Pakistan (data not shown). Jin *et al* reported that most of the currently circulating G1 strains and the Rotarix™ vaccine RRV-S1 strain differ in their antigenic properties and mutations in these antigenic sites may ultimately cause vaccine failure [56].

Despite these variable epidemiological reports, it has been now established that rotavirus vaccines are equally effective against vast diversity of rotavirus genotypes by generating heterotypic immune response. In the recent vaccine trails, both Rotarix™ and RotaTeq™ have been proven as equally effective in regions with high child and adult mortality [57]. Multiple reviews from high and middle income countries have reported a substantial reduction in disease burden within a few years of vaccine implementation through decreased magnitude of rotavirus associated diarrhea and deaths [58], [59]. For instance, studies in Mexico and Brazil reported a reduction in diarrhea related deaths in infants and young children after the introduction of rotavirus vaccine [60], [61]. Rotarix™ efficacy has been evaluated in a large clinical trial of more than 63,000 infants from 11 Latin American countries and Finland, and was found to be safe and highly immunogenic [62], [63]. Similarly, in a randomized, double-blind, placebo-controlled study conducted in 6 European countries, Rotarix™ was observed to be highly immunogenic [64]. RotaTeq™ efficacy has also been evaluated in two phase-III trials among healthy infants, including a large clinical trial of more than 70,000 infants enrolled primarily in the United States and Finland, and found to be highly immunogenic [65], [66]. As a final point, in April 2009, the World Health Organization’s (WHO) Strategic Advisory Group of Experts (SAGE) on immunization recommended the inclusion of rotavirus vaccination of infants into all national immunization programs, with a stronger recommendation for countries where “diarrheal deaths account for ≥10% of mortality among children aged <5 years” [67]. In addition, it has been emphasized that the introduction of rotavirus vaccine should be accompanied by measures to ensure high vaccination and timely administration of each vaccine dose.

### Conclusion

Our present findings conclude that the rotavirus genotypes circulating in different geographical areas of Pakistan are quite variable and large scale studies must be conducted to calculate the burden of disease as well as the epidemiological understanding of contributing viral genotypes. Similar diversity of prevalent rotavirus strains within the Indian subcontinent including Pakistan has been reported by Miles et al., [41]. The annual birth rate in Pakistan is approximately five million; the right rotavirus vaccine would be greatly helpful to protect newborns from this serious disease and its associated mortality. Although our report does not describe a thorough representation of the prevailing genotypes throughout the country, but it provides significant information for the health policy makers to review and implement informed immunization policy in the country.

## References

[1] Hoshino Y, Kapikian AZ (2000) Rotavirus serotypes: classification and importance in epidemiology, immunity, and vaccine development. J Health Popul Nutr 18: 5–14. 11014764

[2] Rotavirus vaccines. Wkly Epidemiol Rec 82: 285–295. 17691162

[3] Parashar UD, Hummelman EG, Bresee JS, Miller MA, Glass RI (2003) Global illness and deaths caused by rotavirus disease in children. Emerg Infect Dis 9: 565–572. 12737740 10.3201/eid0905.020562 PMC2972763

[4] Glass RI, Bresee JS, Turcios R, Fischer TK, Parashar UD, et al (2005) Rotavirus vaccines: targeting the developing world. J Infect Dis 192 Suppl 1S160–166. 16088799 10.1086/431504

[5] Rotavirus surveillance–worldwide, 2001–2008. MMWR Morb Mortal Wkly Rep 57: 1255–1257. 19023263

[6] Phan TG, Khamrin P, Quang TD, Dey SK, Takanashi S, et al (2007) Detection and genetic characterization of group A rotavirus strains circulating among children with acute gastroenteritis in Japan. J Virol 81: 4645–4653. 17301134 10.1128/JVI.02342-06 PMC1900178

[7] Matthijnssens J, Ciarlet M, Rahman M, Attoui H, Banyai K, et al (2008) Recommendations for the classification of group A rotaviruses using all 11 genomic RNA segments. Arch Virol 153: 1621–1629. 18604469 10.1007/s00705-008-0155-1 PMC2556306

[8] Gentsch JR, Laird AR, Bielfelt B, Griffin DD, Banyai K, et al (2005) Serotype diversity and reassortment between human and animal rotavirus strains: implications for rotavirus vaccine programs. J Infect Dis 192 Suppl 1S146–159. 16088798 10.1086/431499

[9] Santos N, Hoshino Y (2005) Global distribution of rotavirus serotypes/genotypes and its implication for the development and implementation of an effective rotavirus vaccine. Rev Med Virol 15: 29–56. 15484186 10.1002/rmv.448

[10] Martella V, Banyai K, Matthijnssens J, Buonavoglia C, Ciarlet M (2010) Zoonotic aspects of rotaviruses. Vet Microbiol 140: 246–255. 19781872 10.1016/j.vetmic.2009.08.028

[11] Matthijnssens J, Bilcke J, Ciarlet M, Martella V, Banyai K, et al (2009) Rotavirus disease and vaccination: impact on genotype diversity. Future Microbiol 4: 1303–1316. 19995190 10.2217/fmb.09.96

[12] Koopmans M, Brown D (1999) Seasonality and diversity of Group A rotaviruses in Europe. Acta Paediatr Suppl 88: 14–19. 10.1111/j.1651-2227.1999.tb14320.x 10088906

[13] Alam MM, Malik SA, Shaukat S, Naeem A, Sharif S, et al (2009) Genetic characterization of rotavirus subtypes in Pakistan-first report of G12 genotype from Pakistan under WHO-Eastern Mediterranean region. Virus Res 144: 280–284. 19720243 10.1016/j.virusres.2009.03.015

[14] Gouvea V, Glass RI, Woods P, Taniguchi K, Clark HF, et al (1990) Polymerase chain reaction amplification and typing of rotavirus nucleic acid from stool specimens. J Clin Microbiol 28: 276–282. 2155916 10.1128/jcm.28.2.276-282.1990 PMC269590

[15] Gentsch JR, Glass RI, Woods P, Gouvea V, Gorziglia M, et al (1992) Identification of group A rotavirus gene 4 types by polymerase chain reaction. J Clin Microbiol 30: 1365–1373. 1320625 10.1128/jcm.30.6.1365-1373.1992 PMC265294

[16] Aoki ST, Settembre EC, Trask SD, Greenberg HB, Harrison SC, et al (2009) Structure of rotavirus outer-layer protein VP7 bound with a neutralizing Fab. Science 324: 1444–1447. 19520960 10.1126/science.1170481 PMC2995306

[17] Monnier N, Higo-Moriguchi K, Sun ZY, Prasad BV, Taniguchi K, et al (2006) High-resolution molecular and antigen structure of the VP8* core of a sialic acid-independent human rotavirus strain. J Virol 80: 1513–1523. 16415027 10.1128/JVI.80.3.1513-1523.2006 PMC1346936

[18] Lopman BA, Reacher MH, Van Duijnhoven Y, Hanon FX, Brown D, et al (2003) Viral gastroenteritis outbreaks in Europe, 1995–2000. Emerg Infect Dis 9: 90–96. 12533287 10.3201/eid0901.020184 PMC2873740

[19] McIver CJ, Hansman G, White P, Doultree JC, Catton M, et al (2001) Diagnosis of enteric pathogens in children with gastroenteritis. Pathology 33: 353–358. 11523939

[20] Simpson R, Aliyu S, Iturriza-Gomara M, Desselberger U, Gray J (2003) Infantile viral gastroenteritis: on the way to closing the diagnostic gap. J Med Virol 70: 258–262. 12696113 10.1002/jmv.10386

[21] Dennehy PH (2005) Acute diarrheal disease in children: epidemiology, prevention, and treatment. Infect Dis Clin North Am 19: 585–602. 16102650 10.1016/j.idc.2005.05.003

[22] Logan C, O’Leary JJ, O’Sullivan N (2006) Real-time reverse transcription-PCR for detection of rotavirus and adenovirus as causative agents of acute viral gastroenteritis in children. J Clin Microbiol 44: 3189–3195. 16954246 10.1128/JCM.00915-06 PMC1594742

[23] Iturriza-Gomara M, Green J, Brown DW, Ramsay M, Desselberger U, et al (2000) Molecular epidemiology of human group A rotavirus infections in the United Kingdom between 1995 and 1998. J Clin Microbiol 38: 4394–4401. 11101570 10.1128/jcm.38.12.4394-4401.2000 PMC87611

[24] Kawai K, O’Brien MA, Goveia MG, Mast TC, El Khoury AC (2012) Burden of rotavirus gastroenteritis and distribution of rotavirus strains in Asia: a systematic review. Vaccine 30: 1244–1254. 22212128 10.1016/j.vaccine.2011.12.092

[25] Ansari SA, Springthorpe VS, Sattar SA (1991) Survival and vehicular spread of human rotaviruses: possible relation to seasonality of outbreaks. Rev Infect Dis 13: 448–461. 1866549 10.1093/clinids/13.3.448

[26] Bishop RF (1996) Natural history of human rotavirus infection. Arch Virol Suppl 12: 119–128. 10.1007/978-3-7091-6553-9_14 9015109

[27] Stoll BJ, Glass RI, Huq MI, Khan MU, Holt JE, et al (1982) Surveillance of patients attending a diarrhoeal disease hospital in Bangladesh. Br Med J (Clin Res Ed) 285: 1185–1188. 10.1136/bmj.285.6349.1185 PMC1500105 6812801

[28] Bresee J, Fang ZY, Wang B, Nelson EA, Tam J, et al (2004) First report from the Asian Rotavirus Surveillance Network. Emerg Infect Dis 10: 988–995. 15207047 10.3201/eid1006.030519 PMC3323142

[29] Dutta SR, Khalfan SA, Baig BH, Philipose L, Fulayfil R (1990) Epidemiology of rotavirus diarrhoea in children under five years in Bahrain. Int J Epidemiol 19: 722–727. 2175734 10.1093/ije/19.3.722

[30] Hieber JP, Shelton S, Nelson JD, Leon J, Mohs E (1978) Comparison of human rotavirus disease in tropical and temperate settings. Am J Dis Child 132: 853–858. 210656 10.1001/archpedi.1978.02120340029004

[31] Glass RI, Kilgore PE, Holman RC, Jin S, Smith JC, et al (1996) The epidemiology of rotavirus diarrhea in the United States: surveillance and estimates of disease burden. J Infect Dis 174 Suppl 1S5–11. 8752284 10.1093/infdis/174.supplement_1.s5

[32] Konno T, Suzuki H, Katsushima N, Imai A, Tazawa F, et al (1983) Influence of temperature and relative humidity on human rotavirus infection in Japan. J Infect Dis 147: 125–128. 6822748 10.1093/infdis/147.1.125

[33] Nakagomi T, Nakagomi O, Takahashi Y, Enoki M, Suzuki T, et al (2005) Incidence and burden of rotavirus gastroenteritis in Japan, as estimated from a prospective sentinel hospital study. J Infect Dis 192 Suppl 1S106–110. 16088792 10.1086/431503

[34] Bishop RF, Masendycz PJ, Bugg HC, Carlin JB, Barnes GL (2001) Epidemiological patterns of rotaviruses causing severe gastroenteritis in young children throughout Australia from 1993 to 1996. J Clin Microbiol 39: 1085–1091. 11230431 10.1128/JCM.39.3.1085-1091.2001 PMC87877

[35] Cook SM, Glass RI, LeBaron CW, Ho MS (1990) Global seasonality of rotavirus infections. Bull World Health Organ 68: 171–177. 1694734 PMC2393128

[36] Qazi R, Sultana S, Sundar S, Warraich H, un-Nisa T, et al (2009) Population-based surveillance for severe rotavirus gastroenteritis in children in Karachi, Pakistan. Vaccine 27 Suppl 5F25–30. 19931714 10.1016/j.vaccine.2009.08.064

[37] Wen L, Ushijima H, Kakizawa J, Fang ZY, Nishio O, et al (1995) Genetic variation in VP7 gene of human rotavirus serotype 2 (G2 type) isolated in Japan, China, and Pakistan. Microbiol Immunol 39: 911–915. 8657020 10.1111/j.1348-0421.1995.tb03277.x

[38] Nishio O, Matsui K, Oka T, Ushijima H, Mubina A, et al (2000) Rotavirus infection among infants with diarrhea in Pakistan. Pediatr Int 42: 425–427. 10986882 10.1046/j.1442-200x.2000.01256.x

[39] Bhan MK, Lew JF, Sazawal S, Das BK, Gentsch JR, et al (1993) Protection conferred by neonatal rotavirus infection against subsequent rotavirus diarrhea. J Infect Dis 168: 282–287. 8393054 10.1093/infdis/168.2.282

[40] Clark HF, Lawley DA, Schaffer A, Patacsil JM, Marcello AE, et al (2004) Assessment of the epidemic potential of a new strain of rotavirus associated with the novel G9 serotype which caused an outbreak in the United States for the first time in the 1995–1996 season. J Clin Microbiol 42: 1434–1438. 15070985 10.1128/JCM.42.4.1434-1438.2004 PMC387540

[41] Miles MG, Lewis KD, Kang G, Parashar UD, Steele AD (2012) A systematic review of rotavirus strain diversity in India, Bangladesh, and Pakistan. Vaccine 30 Suppl 1A131–139. 22520122 10.1016/j.vaccine.2011.10.002

[42] Rahman M, Matthijnssens J, Nahar S, Podder G, Sack DA, et al (2005) Characterization of a novel P[25],G11 human group a rotavirus. J Clin Microbiol 43: 3208–3212. 16000437 10.1128/JCM.43.7.3208-3212.2005 PMC1169153

[43] Das S, Varghese V, Chaudhury S, Barman P, Mahapatra S, et al (2003) Emergence of novel human group A rotavirus G12 strains in India. J Clin Microbiol 41: 2760–2762. 12791925 10.1128/JCM.41.6.2760-2762.2003 PMC156500

[44] Rotavirus vaccines. WHO position paper - January 2013. Wkly Epidemiol Rec 88: 49–64. 23424730

[45] McDonald SM, Matthijnssens J, McAllen JK, Hine E, Overton L, et al (2009) Evolutionary dynamics of human rotaviruses: balancing reassortment with preferred genome constellations. PLoS Pathog 5: e1000634. 19851457 10.1371/journal.ppat.1000634 PMC2760143

[46] Zeller M, Rahman M, Heylen E, De Coster S, De Vos S, et al (2010) Rotavirus incidence and genotype distribution before and after national rotavirus vaccine introduction in Belgium. Vaccine 28: 7507–7513. 20851085 10.1016/j.vaccine.2010.09.004

[47] Zeller M, Patton JT, Heylen E, De Coster S, Ciarlet M, et al (2012) Genetic analyses reveal differences in the VP7 and VP4 antigenic epitopes between human rotaviruses circulating in Belgium and rotaviruses in Rotarix and RotaTeq. J Clin Microbiol 50: 966–976. 22189107 10.1128/JCM.05590-11 PMC3295124

[48] Kirkwood CD, Boniface K, Barnes GL, Bishop RF (2011) Distribution of rotavirus genotypes after introduction of rotavirus vaccines, Rotarix(R) and RotaTeq(R), into the National Immunization Program of Australia. Pediatr Infect Dis J 30: S48–53. 21183840 10.1097/INF.0b013e3181fefd90

[49] Boom JA, Tate JE, Sahni LC, Rench MA, Hull JJ, et al (2010) Effectiveness of pentavalent rotavirus vaccine in a large urban population in the United States. Pediatrics 125: e199–207. 20083525 10.1542/peds.2009-1021

[50] Hull JJ, Teel EN, Kerin TK, Freeman MM, Esona MD, et al (2011) United States rotavirus strain surveillance from 2005 to 2008: genotype prevalence before and after vaccine introduction. Pediatr Infect Dis J 30: S42–47. 21183839 10.1097/INF.0b013e3181fefd78

[51] Iturriza-Gomara M, Cubitt D, Steele D, Green J, Brown D, et al (2000) Characterisation of rotavirus G9 strains isolated in the UK between 1995 and 1998. J Med Virol 61: 510–517. 10897071 10.1002/1096-9071(200008)61:4<510::aid-jmv15>3.0.co;2-q

[52] Oka T, Nakagomi T, Nakagomi O (2000) Apparent re-emergence of serotype G9 in 1995 among rotaviruses recovered from Japanese children hospitalized with acute gastroenteritis. Microbiol Immunol 44: 957–961. 11145279 10.1111/j.1348-0421.2000.tb02590.x

[53] Laird AR, Gentsch JR, Nakagomi T, Nakagomi O, Glass RI (2003) Characterization of serotype G9 rotavirus strains isolated in the United States and India from 1993 to 2001. J Clin Microbiol 41: 3100–3111. 12843049 10.1128/JCM.41.7.3100-3111.2003 PMC165321

[54] Coulson BS, Kirkwood C (1991) Relation of VP7 amino acid sequence to monoclonal antibody neutralization of rotavirus and rotavirus monotype. J Virol 65: 5968–5974. 1656083 10.1128/jvi.65.11.5968-5974.1991 PMC250261

[55] Gomara MI, Cubitt D, Desselberger U, Gray J (2001) Amino acid substitution within the VP7 protein of G2 rotavirus strains associated with failure to serotype. J Clin Microbiol 39: 3796–3798. 11574622 10.1128/JCM.39.10.3796-3798.2001 PMC88438

[56] Jin Q, Ward RL, Knowlton DR, Gabbay YB, Linhares AC, et al (1996) Divergence of VP7 genes of G1 rotaviruses isolated from infants vaccinated with reassortant rhesus rotaviruses. Arch Virol 141: 2057–2076. 8973523 10.1007/BF01718215

[57] Soares-Weiser K, Maclehose H, Ben-Aharon I, Goldberg E, Pitan F, et al.. (2010) Vaccines for preventing rotavirus diarrhoea: vaccines in use. Cochrane Database Syst Rev: CD008521. 10.1002/14651858.CD008521 20464766

[58] Patel MM, Clark AD, Sanderson CF, Tate J, Parashar UD (2012) Removing the age restrictions for rotavirus vaccination: a benefit-risk modeling analysis. PLoS Med 9: e1001330. 23109915 10.1371/journal.pmed.1001330 PMC3479108

[59] Giaquinto C, Dominiak-Felden G, Van Damme P, Myint TT, Maldonado YA, et al (2011) Summary of effectiveness and impact of rotavirus vaccination with the oral pentavalent rotavirus vaccine: a systematic review of the experience in industrialized countries. Hum Vaccin 7: 734–748. 21734466 10.4161/hv.7.7.15511

[60] Richardson V, Hernandez-Pichardo J, Quintanar-Solares M, Esparza-Aguilar M, Johnson B, et al (2010) Effect of rotavirus vaccination on death from childhood diarrhea in Mexico. N Engl J Med 362: 299–305. 20107215 10.1056/NEJMoa0905211

[61] do Carmo GM, Yen C, Cortes J, Siqueira AA, de Oliveira WK, et al (2011) Decline in diarrhea mortality and admissions after routine childhood rotavirus immunization in Brazil: a time-series analysis. PLoS Med 8: e1001024. 21526228 10.1371/journal.pmed.1001024 PMC3079643

[62] Linhares AC, Velazquez FR, Perez-Schael I, Saez-Llorens X, Abate H, et al (2008) Efficacy and safety of an oral live attenuated human rotavirus vaccine against rotavirus gastroenteritis during the first 2 years of life in Latin American infants: a randomised, double-blind, placebo-controlled phase III study. Lancet 371: 1181–1189. 18395579 10.1016/S0140-6736(08)60524-3

[63] Ruiz-Palacios GM, Perez-Schael I, Velazquez FR, Abate H, Breuer T, et al (2006) Safety and efficacy of an attenuated vaccine against severe rotavirus gastroenteritis. N Engl J Med 354: 11–22. 16394298 10.1056/NEJMoa052434

[64] Vesikari T, Karvonen A, Puustinen L, Zeng SQ, Szakal ED, et al (2004) Efficacy of RIX4414 live attenuated human rotavirus vaccine in Finnish infants. Pediatr Infect Dis J 23: 937–943. 15602194 10.1097/01.inf.0000141722.10130.50

[65] Block SL, Vesikari T, Goveia MG, Rivers SB, Adeyi BA, et al (2007) Efficacy, immunogenicity, and safety of a pentavalent human-bovine (WC3) reassortant rotavirus vaccine at the end of shelf life. Pediatrics 119: 11–18. 17200266 10.1542/peds.2006-2058

[66] Vesikari T, Matson DO, Dennehy P, Van Damme P, Santosham M, et al (2006) Safety and efficacy of a pentavalent human-bovine (WC3) reassortant rotavirus vaccine. N Engl J Med 354: 23–33. 16394299 10.1056/NEJMoa052664

[67] Meeting of the immunization Strategic Advisory Group of Experts, April 2009–conclusions and recommendations. Wkly Epidemiol Rec 84: 220–236. 19499606

