# Correction: Epidemiology and Genetic Diversity of Rotavirus Strains in Children with Acute Gastroenteritis in Lahore, Pakistan

**DOI:** 10.1371/annotation/68a1d471-b3b1-45e7-9b81-d242f1c20ad1

**Published:** 2013-12-03

**Authors:** Muhammad Masroor Alam, Adnan Khurshid, Shahzad Shaukat, Rana Muhammad Suleman, Salmaan Sharif, Mehar Angez, Salman Akbar Malik, Tahir Masood Ahmed, Uzma Bashir Aamir, Muhammad Naeem, Syed Sohail Zahoor Zaidi

The titles and legends for Figures 1 and 2 were incorrectly switched. The title and legend currently appearing with Figure 1 belong with Figure 2, and the title and legend currently appearing with Figure 2 belong with Figure 1. The images themselves are in correct order. 

